# Potential of Bio-Sourced Oligogalacturonides in Crop Protection

**DOI:** 10.3390/molecules30061392

**Published:** 2025-03-20

**Authors:** Camille Carton, Maryline Magnin-Robert, Béatrice Randoux, Corinne Pau-Roblot, Anissa Lounès-Hadj Sahraoui

**Affiliations:** 1 Unité de Chimie Environnementale et Interactions sur le Vivant (UCEIV)—UR 4492, Université du Littoral Côte d’Opale, 50 Rue Ferdinand Buisson, 62228 Calais Cedex, France; camille.carton@u-picardie.fr (C.C.); maryline.magnin-robert@univ-littoral.fr (M.M.-R.); beatrice.randoux@univ-littoral.fr (B.R.); 2 UMRT INRAE 1158 BioEcoAgro—Biologie des Plantes et Innovation, Université de Picardie Jules Verne, UFR des Sciences, 33 Rue St Leu, 80039 Amiens, France; corinne.pau-roblot@u-picardie.fr

**Keywords:** pectins, oligogalacturonides, biocontrol, by-product

## Abstract

During plant development or interactions with pathogens, modifications of the plant cell wall occur. Among the enzymes involved, pectinases, particularly polygalacturonases (PGases), play a crucial role in the controlled hydrolysis of cell wall polysaccharides, leading to the formation of oligogalacturonides (OGs). These pectin-derived fragments act as key elicitors of plant defense responses, stimulating innate immunity and enhancing resistance to pathogens by modulating the expression of genes involved in immune responses and inducing the production of defense compounds. OGs are of particular interest for plant protection as a natural alternative to conventional phytosanitary products as they can be obtained through chemical, thermal, or enzymatic degradation of plant biomass. In a sustainable approach, agricultural by-products rich in pectin, such as citrus peels, apple pomace, or sugar beet pulp, offer an eco-friendly and cost-effective alternative for OG production. Thus, the current review aims to (i) update the state of the art about the different methods used to produce OGs, (ii) explore the potential of OGs as bio-based biocontrol molecules, and (iii) examine the relevance of new pectin sources for OG production.

## 1. Introduction

Due to the growing global demand for agricultural products, traditional farming systems have increasingly shifted toward intensive production models that heavily rely on chemical fertilizers and pesticides, which are harmful to both the environment and human health [1,2]. In the context of agroecological transition, developing alternative solutions to gradually reduce the use of chemical-based phytosanitary products has become a key priority for the future of agriculture. This transition aims to promote a more sustainable agricultural model that preserves environmental integrity and human health while maintaining productivity levels.

To tackle these complex challenges, (i) adapting to climate change while ensuring sustainable resource management, (ii) transitioning to sustainable agriculture by reducing chemical inputs and promoting regenerative practices, and (iii) enhancing food sovereignty by strengthening local food systems and farmers’ autonomy, it is essential to adopt new approaches. Global and European governmental initiatives, such as the Farm to Fork strategy [3], have been implemented. These initiatives underline the importance of innovation in the development of new biocontrol products for more eco-friendly agricultural systems. Advances in biocontrol solutions will play an essential role in implementing sustainable agricultural practices to meet the demands of modern food production systems.

Biocontrol is based on the use of natural biological mechanisms to control pests and plant diseases. Among the tools of biocontrol, plant defense inducers (called also elicitors) represent a promising alternative [4]. They act by eliciting natural plant defense reactions and improving plant resistance against biotic stresses (pathogens and insects) [5].

General elicitors act differently depending on the cultivar within a plant species and play a crucial role in primary innate immunity. These elicitors include chemical compounds, microbe-associated molecular patterns (MAMPs) derived from non-pathogenic microorganisms, damage-associated molecular patterns (DAMPs) resulting from the degradation of the plant cell wall due to the pathogen’s activity, and pathogen-associated molecular patterns (PAMPs) originating from pathogenic microorganisms [6,7]. Although the perception of elicitors is generally receptor-dependent, only a limited number of binding sites have been characterized to date [8,9]. This highlights the complexity of plant immune responses and the need for further research to elucidate the molecular mechanisms underlying elicitor recognition.

Among natural elicitors, derivatives from the plant cell wall have been shown to play a crucial role in plant defense [10]. Oligogalacturonides (OGs), obtained from the degradation of the cell wall pectins, are composed of α-(1→4)-linked galacturonic acid (GalA) oligomers [11,12]. OGs can be generated during pathogen infection through the enzymatic activity of hydrolytic proteins, such as polygalacturonases (PGases), pectate lyases (PLases), and pectin lyases (PNLases). These enzymes degrade the plant cell wall, which serves as an extracellular source of DAMPs by releasing polysaccharide fragments, including cellobiose and cellotriose derived from cellulose, mixed-linkage glucans, and OGs derived from pectin [13]. The application of OGs on various plants as a preventive treatment against biotic stress has demonstrated their ability to trigger defense responses, such as the accumulation of reactive oxygen species (ROS) [14,15] and the production of defense hormones, including ethylene (ET), salicylic acid (SA), and jasmonic acid (JA) [10,16,17]. These responses also involve the phosphorylation of Mitogen-Activated Protein Kinases (MAPKs) [18,19] and the activation of enzymes involved in plant defense, such as peroxidase (POX), lipoxygenase (LOX), or catalase (CAT) [20,21].

To date, several methods for degrading pectins (mostly commercial pectins) have been employed to produce OGs. Three categories can be distinguished as (i) chemical degradation [22], (ii) thermal degradation, which involves heating pectin at high temperatures, sometimes combined with chemical esterification to produce esterified OGs, as described in the studies of Randoux et al. (2010) and Selim et al. (2017) [21,23], and (iii) enzymatic hydrolysis, which is used to produce OGs with various methyl and/or acetyl-esterified groups [24].

Agriculture and the agri-food industry generate large amounts of plant residues, many of which are rich in pectins. However, the potential use of these residues to produce OGs remains largely unexplored. To date, only limited research has focused on the valorization of agricultural and agri-food by-products for OG production. Thus, developing a non-chemical process to produce OGs while simultaneously recycling pectin-rich waste represents a promising approach for a more sustainable agriculture. Indeed, the OGs obtained through this method would be both biodegradable and non-toxic, offering an eco-friendly alternative for agricultural applications [25].

The present review aims to (i) update the state of the art concerning the different methods used to produce OGs, (ii) explore the potential of OGs as bio-based biocontrol molecules, and (iii) examine the relevance of new pectin sources for OG production.

## 2. From Plant Cell Wall to Oligogalacturonides (OGs)

OGs are naturally produced by plants in response to pathogen infection. Indeed, when the pathogen agents interact with the host plant, they secrete pectin-modifying enzymes that break down the homogalacturonan chains of pectin in the plant cell wall, resulting in OGs’ release [26]. These OGs have proven to be interesting molecules for human health [27] and particularly promising for plant protection [10].

The need to produce OGs for several applications has led to the development of different (chemical, thermal, and enzymatic) pectin degradation approaches.

### 2.1. OGs’ Chemical Structure

**Figure 1:**
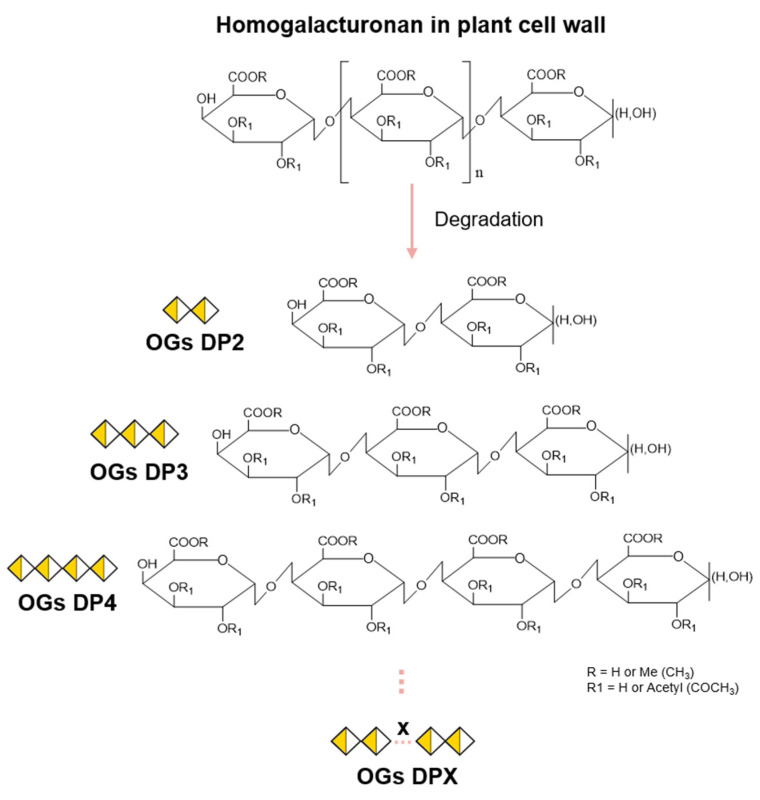
Formation of oligogalacturonides from a homogalacturonan chain. Oligoglacturonides (OGs) are formed following hydrolysis of the homogalacturonan (HG) chain. The degree of polymerization (DP) will vary according to HG cleavage sites, producing OGs from DP2 to DPX. Depending on the pectic source used prior to hydrolysis, methylesterified (R) and/or acetylesterified (R1) groups may be present on the OGs produced, thus varying the degree of methylation (DM) or acetylation (DA).

OGs are galacturonic acid oligomers linked in α-(1→4) resulting from the degradation of the plant cell wall (Figure 1). Typical plant cell walls are distinguished by the presence of layers of varying compositions: the middle lamella, which acts as an intercellular adhesive, maintaining cell cohesion, primary cell walls, which surround growing cells, and secondary cell walls, which envelop specialized cells with thicker structures, such as vascular elements or fibrous cells [12,28,29]. The structure and composition of cell walls vary according to plant species, tissue, age, and cell type [30].

For the production of OGs, the primary cell wall is of particular interest. The latter is a scaffold of polysaccharide compound (90%) in a soluble protein matrix (10%). The main polysaccharide is cellulose (≈30%), and the other polysaccharides are grouped into two categories: (i) pectic polysaccharides (≈35%) comprising homogalacturonan, xylogalacturonan, and rhamnogalacturonan I and II [31] and (ii) hemicellulosic polysaccharides comprising xyloglucans, glucomannans, xylans, and glucans [29,32,33].

The production of OGs depends on the presence of pectin and, more specifically, of GalA, which makes up around 70% of pectins in most plants. Although all pectic polysaccharides contain this GalA, they can exhibit widely varying structures regarding the composition of other sugar constituents [34,35,36]. In the plant cell wall, homogalacturonan (HG) is the most abundant pectic polysaccharide (∼55–70%) [37,38]. HG is a linear chain of α-(1→4)-linked GalA that can be methylesterified at C-6 and/or O-acetylated at O2/O3 (Figure 1) [29,34,38].

**Figure 2:**
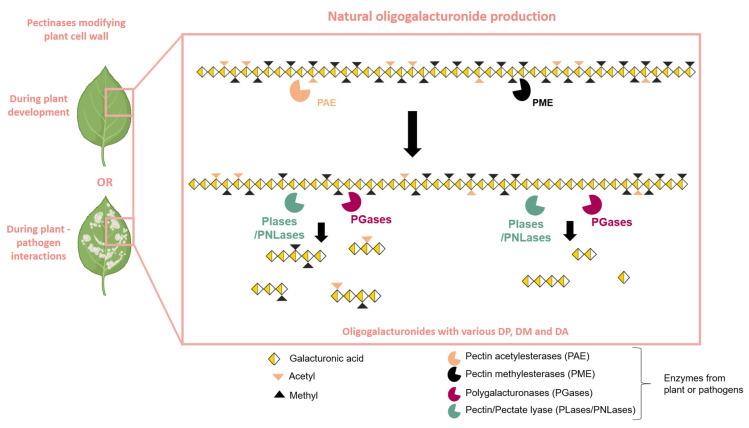
Natural production of oligogalacturonides by plants or pathogen pectinases. Plant cell wall modifications can occur during plant development or during plant–pathogen interactions. Pectinolytic enzymes, such as PAE (pectin acetylesterase) and PME (pectin methylesterase), prepare the homogalacturonan chain for hydrolysis by PLase/PNLase (Pectin/Pectate lyase) or PGase (polygalacturonase). The action of PLase/PNLase and PGase releases oligogalacturonides (OGs) with varying degrees of polymerization (DP), methylation (DM), and acetylation (DA).

Thus, OGs are produced through the partial hydrolysis of the HG chain through the action of various HG-modifying enzymes, which occur naturally during plant–pathogen interactions [10] or during plant development [39]. The action of pectin methyl esterases (PMEs) and pectin acetyl esterases (PAEs) facilitates the blockwise or random de-esterification of homogalacturonan (HG) chains [40,41] (Figure 2). The combined activity of PMEs and PAEs generates substrates for PGases as well as for pectate or pectin lyases (PLase/PNLase) [39]. PGases are among the most extensively studied pectinolytic enzymes, belonging to the glycoside hydrolase family and containing at least one GH28 domain [42]. Depending on their mode of action, PGases fall into two main categories: endo-PGases (E.C. 3.2.1.15) and exo-PGases (E.C. 3.2.1.67). Endo-PGases cleave the α-(1→4) bonds within the polygalacturonan chain, while exo-PGases attack the chain from its end with hydrolysis of the glycosidic bonds on the non-reducing side of the GalA residues [39,43]. The HG chain is naturally hydrolyzed to obtain OGs by PGases and particularly endo-PGases, PLase, or PNLase with plant or pathogen origins [26] (Figure 2).

The HG chain is characterized by the degree of polymerization (DP), the degree of methylation (DM) (number of methyl groups), and the degree of acetylation (DA) (number of acetyl groups). Thus, the site of action of the HG-modifying enzymes will depend on the structure of the HG chain prior to hydrolysis and may vary according to the DP, DM, and DA. This will result in the production of OGs with different structures (Figure 1).

### 2.2. OG Production Methods

Although the enzymatic pathway is the natural mechanism for OG production in plants, various industrial methods have been developed to produce OGs in large quantities in order to study their role in plant development and protection against plant diseases. The three most commonly used methods for producing OGs, namely chemical, thermal, and enzymatic approaches (Table 1), have recently been described by Martínez-Gómez et al. (2023) [27]. However, mechanical extraction, such as the use of ultrasound on pectins, is also used to obtain OGs [44]. Here, we provide additional data on the different production methods.

#### 2.2.1. Chemical Approach

OG production can be achieved, in controlled conditions, through the chemical degradation of pectins into oligomers. Two conventional methods can be used, acid hydrolysis or chemical oxidation, as described in Table 1 (the chemical approach part).

(i) Acid hydrolysis involves the use of acids to break the α-(1→4) glycosidic bonds of the pectin. For example, Coenen et al. (2008) hydrolysed apple pulp with HCl and TFA to obtain OGs DP 1–10 [45]. OGs DP 2–30 were produced under acidic conditions with nitric acid (pH1.5) from apple juice by-product [46]. A low concentration of acid (0.2% *w*/*w* citric acid or 0.27% *w*/*w* malic acid) with a constant pH of 2.6 and high temperatures (ranging from 100 to 135 °C with a reaction time between 10 and 120 min) were favorable for producing galacturonic acid polymers and decomposing it into fragments of varying sizes with DP 2–14 [47].

(ii) Chemical oxidation uses oxidizing agents, such as cupric acetate, to degrade pectin. Elboutachfaiti et al. (2008) [48] reported that the scission of free hydroxyl radicals from polygalacturonic acid (PGA) carried out in H_2_O_2_ with copper (II) generated 47% small OGs (DP 2–6) in 1 h. Also, the oxidation of apple pectin under the action of H_2_O_2_ (1.25%) and an ozone–oxygen mixture at 70 °C for 60 min resulted in a decrease in the molecular mass of pectin from 6.4 to 125 kDa [61]. More recently, the extraction of pectins from pomelo peel under acidic and alkaline conditions followed by oxidative hydrolysis with H_2_O_2_ assisted by microwave has been performed to produce small OGs (DP 2–5) with relatively good yield (>50%) [49].

These methods are fast, efficient, and allow for relatively precise control of reaction conditions (pH, temperature, acid, or oxidant concentration), making them suitable for large-scale production. Additionally, they can generate a diverse range of OGs with varying structures, influencing their biological properties. However, this approach also has notable drawbacks, including the use of potentially toxic oxidizing agents (such as hydrogen peroxide (H_2_O_2_) or metal-based oxidants), which may leave undesirable residues in the final product. Moreover, controlling the selectivity of oxidation can be challenging, leading to the formation of unwanted by-products and requiring additional purification steps. Finally, the environmental impact of waste generated by these chemical processes is a concern. That is why enzymatic approaches seem to be more attractive from an ecological standpoint.

#### 2.2.2. Thermal Approach

Thermal degradation of pectin, occurring between 60 and 200 °C (Table 1, thermal approach part), involves the application of heat to break the glycosidic bonds of the pectic chain, leading to the release of OGs.

Einhorn-Stoll et al. (2020) showed that the thermal degradation of citrus pectin at 60 °C under controlled humidity conditions (40% or 80%) induces the depolymerization of pectin, leading to the formation of OGs. The DM of these OGs decreases further over time (up to 28 days) and under higher relative humidity (80%) [50]. Moreover, OGs with a DP ranging from 2 to 25 were obtained through thermal degradation of PGA at 121 °C, one bar, for 40 min [21,62]. In addition to the thermal treatment, chemical acetylation was performed using acetic anhydride to produce OGs with a DA of 25% [21]. Miyazawa et al. (2008) have also produced OGs from PGA with a DP between 1 and 10 by carrying out heat treatment between 160 and 240 °C [53].

Another method used nitric acid followed by electron beam treatment to extract pectin from grapefruit and allowed for the production of OGs with a DP 9 [54]. Furthermore, the combination of sequential aqueous extraction and hydrothermal treatment resulted in the formation of OGs from orange peel waste [22]. Orange polygalacturonic acid was also used to produce OGs with DP four to six after 100 °C heat treatment [52].

These methods have the advantage of not requiring specific enzymes or specific chemical products, which can be beneficial in terms of process cost and simplicity. However, the need for a high temperature consumes a lot of energy.

#### 2.2.3. Enzymatic Approach

The enzymatic production of OGs using PGases is the most interesting method for obtaining OG mixtures with a wide range of DP under gentler growing conditions (Table 1, enzymatic approach part). PGases, belonging to the glycosyl hydrolase 28 family, have the ability to hydrolyze α-(1→4) linkages of the polygalacturonic acid chain releasing, OGs or galacturonic acid monomers [42]. In plants, PGases participate in various processes, like fruit maturation and development [63,64,65], as well as cell elongation or separation [56,66]. They are also involved in plant–pathogen interaction [67].

For example, to produce OG mixtures with DP ranging from 10 to 15 from PGA, a commercial endo-PGases from *Aspergillus niger* (Sigma, Saint-Louis, MO, USA) can be used [58]. This endo-PGase from *A. niger* was also used to produce OGs DP 3 to 18 through the hydrolysis of PGA [59]. Endo-PGases can also be produced in heterologous systems, such as *Pichia pastoris*, as demonstrated by Yang et al. (2021) with AnPG28A from *A. niger*, generating OGs with DP 2–10 through PGA hydrolysis [60]. The advantage of producing PGases in a heterologous system is that it allows for greater diversity in the choice of PGases used during enzymatic hydrolysis. So, in addition to commercial PGases (often from fungi), plant PGases can be used. Thus, Safran et al. (2023) used plant PGases to obtain OGs DP 1–10 and DP 1–9, with various esterification degrees. They hydrolysed commercial citrus pectin (DM 20–34%, Sigma, Saint-Louis, USA) using two plant-derived endo-PGases, AtPGLR and AtADPG2, respectively, produced in heterologous systems of *P. pastoris* [56]. Other enzyme-modifying pectins, such as PLases/PNLases, are rarely used for OG production. However, their use still seems relevant. For example, Voxeur et al. (2019) demonstrated that during the *B. cinerea–A. thaliana* interaction, OGs DP 3 to 10 with various esterifications was released by PNLases. Moreover, 80% of the OGs were generated through the hydrolytic activity of fungal PNLases [68]. Another study also showed that, esterified OGs DP 2 to 7 could be produced by hydrolysis of various pectin sources (citrus pectin DM 24–30% and sugar beet pectins DM 42% DA 31%) by PLase from *Verticillium dahliae* (VdPeIB) [57].

In addition to being non-abrasive for the pectin (preservation of natural esterification of pectins), the enzymatic method enables the production of a more diverse mixture of OGs. Furthermore, when applied to an esterified pectin, as in the study by Safran et al. (2023) [56,57], the enzymatic approach facilitates the generation of esterified OGs, which may exhibit additional biological properties. Lastly, unlike other methods, this approach generates OGs that more closely resemble to those produced during plant–pathogen interactions, thereby enhancing their DAMP properties [68].

In conclusion, whereas chemical and thermal processes mainly allow the production of non-esterified OGs with various DPs (ranging from DP 1 to DP 25), the enzymatic approach leads to the obtention of naturally esterified OGs.

## 3. Role of Oligogalacturonides (OGs) in the Plant’s Immune System Activation

**Figure 3:**
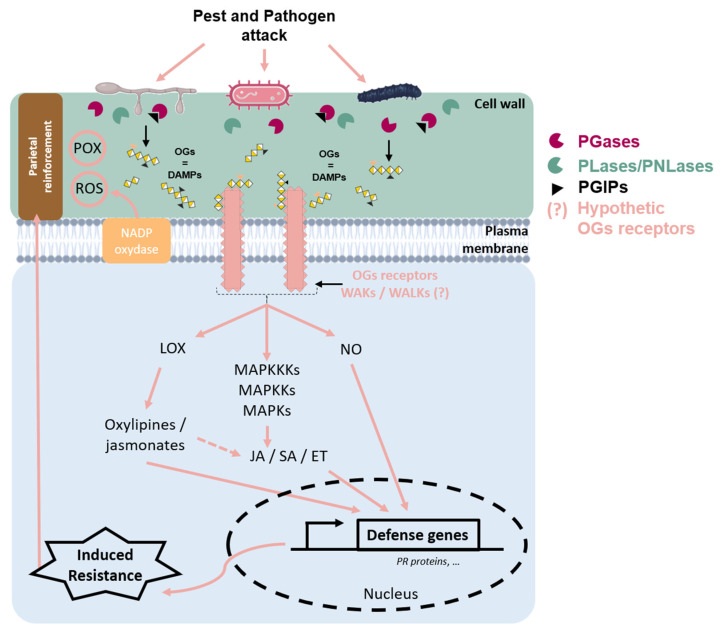
Schematic representation of cell wall events during a pathogen attack on plant tissue. Upon pathogen attack, plant cell wall integrity is disrupted, leading to the release of oligogalacturonides (OGs), which act as damage-associated molecular patterns (DAMPs). These OGs are recognized by putative Wall-Associated Kinases’ (WAKs/WALKs) receptors, triggering intracellular defense signaling. During infection, pathogens secrete polygalacturonases (PGases) to degrade the plant cell wall; however, plant-produced polygalacturonase-inhibiting proteins (PGIPs) limit this degradation and regulate OG release, thereby modulating the immune response. OGs’ perception induces the oxidative burst through the activation of the NADPH–oxidase located in the plasma membrane, leading to the accumulation of reactive oxygen species (ROS), such as H_2_0_2_. It can display antimicrobial activity and contribute to the defense signal and cell wall reinforcement through POX activity. Additionally, OGs’ signaling activates the mitogen-activated protein kinase (MAPK) cascade, which amplifies defense responses and promotes the production of nitric oxide (NO), an important signaling molecule in plant immunity. The octadecanoid pathway can be stimulated, leading to oxylipin and jasmonate production via LOX activity. These defense pathways converge on the induction of defense genes, including those encoding pathogenesis-related (PR) proteins, through the regulation of plant hormones, such as salicylic acid (SA), jasmonic acid (JA), or ethylene (ET). These hormones orchestrate immune responses against pests and pathogens. Together, these mechanisms enhance plant resistance and strengthen cell wall reinforcement, forming an effective defense barrier against pathogen invasion.

The cell wall is a plant’s first line of defense against its environment [68]. It is made up of complex networks of polysaccharides that offer various functional properties: (i) strength, to resist turgor pressure, and (ii) protection against pathogens or pest attacks (nematode, bacteria, or fungi) (Figure 3).

This protective function represents a preexisting physical barrier, which can serve as a site for reactions leading to a basal immunity. During the initial stages of plant–pathogen interaction, the plant detects pathogen-specific molecular signatures, known as Pathogen-Associated Molecular Patterns (PAMPs), as well as endogenous danger signals released in response to infection, referred to as damage-associated molecular patterns (DAMPs) (Figure 3) [7,68,69]. OGs are categorized as DAMPs [10] because they are naturally produced during the degradation of the cell wall pectins following infection by a pathogen [10]. The ability of OGs and polysaccharide fragments of the plant cell wall to induce plant defense was discovered over 40 years ago [70]. They trigger signaling pathways inducing responses involved in rapid plant defense [71].

The first stage of the plant’s immune response to pathogen attack, through the recognition of attack signals (PAMPs or DAMPs), leads to the establishment of pattern-triggered immunity (PTI), a first level of resistance which is not very specific or intense but can delay or even block infection [72]. Although elicitor (like OG) perception is generally receptor-dependent, few binding sites have been characterized to date. The establishment of plant defense mechanisms during PTI takes place in three stages: perception/recognition of the elicitor by the plant, signal transduction, and induction of plant defense gene expression [73].

### 3.1. OGs’ Perception

The most well-documented receptors for pectic fragments, and more specifically for OGs, are the Wall-Associated Kinases (WAKs) (Figure 3). WAKs are receptor proteins with an intracellular kinase-like domain located in the cytoplasm, as well as an extracellular domain that acts as an epidermal growth factor (EGF)-like receptor and a galacturonan-binding domain [74,75]. In *A. thaliana*, WAK proteins are encoded by the *WAK* gene family, which consists of 5 *WAK* genes and 22 *WAK-Like* (*WAKLs*) genes [76]. In monocots, these gene families are even more extensive; for example, 125 WAK genes have been identified in rice (*Oryza sativa*) [77].

WAKs play a key role in the recognition of DAMPs, such as OGs (Figure 3). They are therefore considered to be extracellular receptors of the Pattern Recognition Receptor (PRR) type, capable of triggering plant immunity.

However, the affinity of OGs for WAKs has recently been questioned. Herold et al. (2024) showed that the application of OGs to wakΔ2 mutants of *A. thaliana* (which lacks all five members of the WAK family) resulted in a reduction in *Botrytis cinerea*-induced lesions [78]. This observation suggests that WAKs are not essential for OG-induced immunity against this fungal pathogen.

The question of the WAK-like (WAKLs) ability to perceive OGs remains open. However, to date, no conclusive proof has been established [79], with the exception of WAKL22/RESISTANCE TO *FUSARIUM OXYSPORUM* 1 and WAKL14 [80,81].

Although the perception mechanisms of OGs are not yet completely well-defined, their perception by the plant leads to the implementation of various defense mechanisms (Figure 3).

### 3.2. Stimulation of Plant Defense Responses by OGs

#### 3.2.1. Plant Defense Mechanisms Induced by OGs

Once OGs are detected, plants activate their defense mechanisms. One of the earliest responses involves the activation of a calcium (Ca^2^⁺)-mediated signaling pathway, leading to the production of ROS, such as H_2_O_2_ [14,15] and nitric oxide (NO) (Figure 3) [82]. This early signaling also plays a critical role in the activation of protein kinases through phosphorylation and dephosphorylation processes [83].

Simultaneously, the production of various plant hormones is observed, promoting the activation of two key kinases, AtMPK3 and AtMPK6, in *A. thaliana* [18,19]. AtMPK6, in particular, is involved in the rapid induction of defense genes and resistance against *B. cinerea*, a necrotrophic pathogen [19].

An accumulation of plant hormones, such as ET, SA, and JA, is rapidly observed. For instance, exogenous application of OGs in *A. thaliana* stimulates these hormonal pathways (Figure 3) [16,17,84]. These hormones, in turn, induce the transcriptional activation of defense-related enzymes, including phenylalanine ammonia-lyase (PAL), a key enzyme in the phenylpropanoid pathway, and various LOX and ACC synthases (ACS) involved in the JA and ET pathways, respectively [18,85]. This suggests that OGs modulate defense responses by inducing gene expression, leading to the production and accumulation of enzymes [78]. In strawberries, the application of OGs led to the accumulation of SA and the activation of pathogenesis-related genes, such as *PR5* (accession number EU289405) (Figure 3) [86]. In alfalfa, OGs with a degree of DP ranging from 7 to 15 stimulated the activity of several defense enzymes, including CAT, superoxide dismutase (SOD), POX, and monodehydroascorbate reductase (MDHAR) [20]. The same type of reaction has also been observed in wheat, with the activation of enzymes like POX and LOX after the application of OGs [21]. More recently, application of OGs DP 10 to 15 induced the production of ROS and ET in *A. thaliana* Col-0 and wakΔ2 mutant [78].

#### 3.2.2. Protection Induced by OGs Against Plant Diseases

DAMPs, through OGs, thus trigger responses that lead to the basal resistance of the plant against the pathogens [19,82,85]. The induction of plant resistance through OG treatment has been observed in numerous plant species and demonstrated effectiveness against a wide range of pathogens with diverse lifestyles (Table 2). Several studies have shown that OGs with various DP, DA, and DM can enhance plant defense responses against both biotrophic and necrotrophic pathogens, including bacteria and fungi (Table 2).

OGs with high DP, ranging from 11 to 15, are effective in inducing defense responses and protecting plants. For example, the application of OGs with a DP 11 on grapevine leaves reduced by 50–65% the lesions caused by *B. cinerea* [87]. Similarly, OGs with a DP between 10 and 15 applied on tomato plants induced a significant reduction in lesions against the same pathogen [58]. Moreover, OGs with DP 10–15 induced responses, such as H_2_O_2_ production and the expression of defense-related genes encoding glutathione-S-transferase (GST) or chitinases [58]. In grapevine, OGs application promoted the expression of pathogenesis-related (PR) genes and chitinases belonging to classes I, III, and IV [87,88].

Greco et al. 2024 [89] extracted OGs with a DP > 6 from olive pomace. These OGs induced overexpression of genes involved in defense signaling, such as MAPK, *WRKY* (*WRKY DNA-BINDING PROTEIN*), or *PAD3* (*PHYTOALEXIN DEFICIENT 3*), in *A. thaliana* [89]. In addition, pre-treatment of *A. thaliana* leaves with these OGs induced a partial reduction of the lesions caused by *B. cinerea* [89]. The same effect was observed in *A. thaliana* and in tomato against *Pseudomonas syringae*, with a reduction of the bacterial growth of 20% and 10%, respectively [89]. Another study used OGs from olive by-product (DP 7–15) and showed that their application induced the release of Ca^2+^ ions involved in parietal defense and reinforcement. Moreover, these OGs also reduced *B. cinerea* lesions in *A. thaliana* leaves [55]. Another study showed that OGs DP 10 to 15 applied on leaf of *A. thaliana* against *B. cinerea* showed a reduction in the lesion size [78].

The treatment of wheat with OGs DP 3 to 18 reduced the lesions observed on the spikelets and stems caused by *Fusarium graminearum* [59]. It was reported that the OGs’ application on the wheat led to the over-expression of various defense genes, including five genes coding for POX [59]. It was also shown that foliar application of OGs on wheat reduced symptoms of powdery mildew caused by *Blumeria graminis f. sp. tritici* by 57% through the induction of ROS and the stimulation of POX and LOX activities [21]. Acetylated OGs appear to play a role in eliciting plant defenses, particularly by promoting the accumulation of phenolic compounds at infection sites. However, their effect on the overall protection rate remains limited [21]. The same OGs, applied on *Pisum sativum* roots, reduced lesion symptoms caused by *Aphanomyces euteiches* and induced the expression of defense genes involved in the ROS pathway [23].

Low DP OGs can also trigger responses even if they are less studied. For instance, OGs’ trimers (DP3) stimulated the gene expression involved in SA and JA biosynthesis, reducing the colony formation of *Pectobacterium carotovorum* in *A. thaliana* [16]. OGs with a DP of 2 to 7 applied to sugar beet roots induced partial resistance against *Rhizoctonia solani* through the overexpression of *POX* and *SOD* genes, reducing pathogen development [24].

It is worth noting that the OGs used in the previous studies generally originated from commercial sources or were produced through the degradation of a commercial substrate, generally PGA, thus yielding non-esterified OGs (Table 2). Only the studies of Randoux et al. (2010) and Selim et al. (2017) investigated the use of chemically acetylated OGs [21,23]. In addition, Voxeur et al. (2019) [68] showed that the OGs produced during *A. thaliana–B. cinerea* interaction were esterified, so it seems important to succeed in producing and testing esterified OGs. It is possible to produce esterified OGs through an enzymatic method using PLases, such as VdPeIB [57], or PGases, such as AtADPG2 [56] or AtPGLR [40].

In conclusion, the application of OGs stimulates plant defense responses through the accumulation of ROS, the overexpression of defense genes, and the activation of several enzymatic activities (POX, CAT, LOX…). OGs are able to induce partial protection in major crops, highlighting their potential use as biocontrol molecules.

## 4. Applications, Limitations, and Sustainable Production Perspectives

OGs present interesting potential for crop protection applications. This review has shown that several pools of OGs with various DPs have the capacity to induce plant defense mechanisms and protect plants against different pathogens. In addition, several studies have investigated the effect of OGs on plant growth. Davidsson et al. (2017) showed that small OGs can induce down-expression of genes involved in the development of Arabidopsis [16]. In contrast, another work reported that the application of OGs up to a certain amount had no deleterious impact on plant growth. For example, the application of OGs (10 to 500 µg/mL) to wheat did not affect the plant’s fresh weight, unlike treatment with chitosan, which impaired growth in a dose-dependent manner [59]. Treatment with OGs showed no impact on fresh weight up to 200 µg/mL in *Arabidopsis* [89]. These latest studies reinforce the value of OGs as a plant defense stimulator. Nevertheless, their use is currently limited by their high production costs. Several studies reported in the current review pointed out that the majority of the described OGs originate from commercial pectins [21,23,58,59] or are purchased directly from suppliers (Elicityl, Crolles, France; Sigma, Saint-Louis, USA) [16,78]. Furthermore, given the wide variety of OGs that can be produced in addition to those that have been shown to have a protective effect on plants, very few DPs are available for purchase. For example, only OGs DP2, DP3, or DP4 are commercialized (EUR 8000–40,000 per gram). According to the conditions defined by Randoux et al. (2010) [21], who recommend, for example, the application of OGs at a concentration of 5 g/L to protect wheat plants against powdery mildew in controlled conditions, the application of OGs under field conditions seems to be compromised by the high costs.

Pectin used to produce OGs is a natural polymer found in the primary cell walls of non-woody plant cells. From this perspective, and in the context of more environmentally friendly agriculture, the use of agri-food by-products as pectic substrates appears promising. Two recent studies have demonstrated the benefits of using olive by-products to produce OGs with an elicitor effect [55,89]. OGs have also been produced from orange and lemon peel or waste without being tested on plants [22,25].

Given the number of by-products available, few have been tested for OG production. By-product selection must be based on their pectin richness in order to optimize OG yield production. For example, citrus, olives, grapes, apple, and sugar beet have been identified as potentially rich sources of pectin. However, the use of their by-products, such as apple pomace [90], grape pomace [91,92], and sugar beet pulp [93], remains largely unexplored.

**Figure 4:**
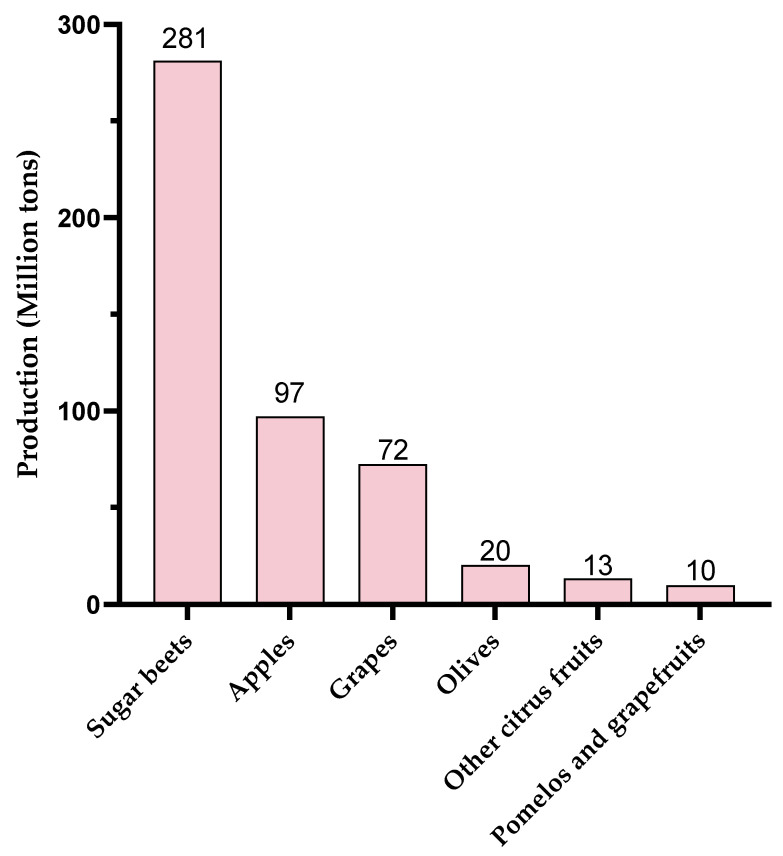
World production (2023) of agricultural products leading to pectin-rich by-products (www.fao.org, accessed on 17 January 2025).

Global production of these different pectic sources is also an important point to consider. In 2023, production of these resources was highly disparate (Figure 4, www.fao.org, accessed on 17 January 2025). World production of olives and citrus fruits was around 20 million tons each. Although OGs with eliciting activity have been produced from olive waste [55,89], this resource does not seem to be the favorite one compared to apple, grape, or sugar beet production. Globally, 72 Mt of grapes, 97 Mt of apples, and 281 Mt of sugar beet were produced in 2023, making them particularly interesting candidates in view of their pectin-rich by-products.

OGs produced from sugar beet, apple, and grapevine by-products appear to be a more suitable approach for large-scale production and cost reduction. However, future studies are needed to assess the ability to produce OGs with consistent chemical structures and biological effects considering different harvested varieties and harvest years worldwide. Enzymatic production could enable the properties of each substrate to be preserved and OGs to be produced in a reproducible way.

Finally, this production could be carried out as part of a circular economy, with the first stage consisting of recovering by-products from farmers and, after processing, giving them back the OGs to protect their crops.

## 5. Conclusions

In plants, OGs are released through the degradation of the homogalacturonan chains making up pectin in the cell wall under the action of hydrolytic enzymes produced by phytopathogens. These enzymes include PMEs, PGases, and PLases/PNLases.

In order to stimulate defense responses in plants, OGs can be applied exogenously. Therefore, they could be produced in different ways, including chemical, thermal, or enzymatic methods.

OGs’ application was proven to stimulate defense responses (ROS accumulation, defense gene overexpression, defense enzyme stimulation) in various plants. Interestingly, it was reported that OG treatments have enabled symptom reductions of many diseases caused by fungal or bacterial pathogens. Moreover, OGs induce partial protection in major and specialized crops, highlighting their potential for expanding the use of these molecules as biocontrol bio-based compounds in the field. In the future, producing OGs from agri-food by-products appears to be a more suitable approach for large-scale and cost-effective production.

## Figures and Tables

**Table 1:** Overview of different methods used to produce OGs. Legends: nd: not determined; Endo-PGase: Endo-polygalacturonase; PGA: polygalacturonic acid; PLase: pectate lyases.

Methods	Pectin Source	Treatment Conditions	Product	References
Chemical	Apple pulp	HCl (48 h, 80 °C) + TFA (6 h, 100 °C)	DP 1–10	[45]
	Orange by-product	Nitric acid (pH 1.5, 120 °C, 0.5 h)	DP 2–30	[46]
	PGA	Citric and malic acid (pH 2.6, 125–135 °C, 10 min to 1 h, 100 bar)	DP 2–7	[47]
	PGA	H_2_O_2_, cupric acetate (1 h, 60 °C)	DP 2–6	[48]
	Pomelo peel pectin	H_2_O_2_ + microwave (0 to 0.15 h)	DP 2–6	[49]
Thermal	Citrus pectin	60 °C for 4 weeks, controlled humidity conditions (40% or 80%)	nd	[50]
	Orange peel wastes	Hydrothermal treatment at 140–200 °C	nd	[22]
	Lemon peel wastes	Hydrothermal treatment at 160 °C	DP 2–8	[51]
	Orange polygalacturonic acid	100 °C—48 h	DP 4–6	[52]
	PGA	pH 4–5, 121 °C, 1 atm, and 40 min	DP 2–25	[21]
	PGA	Hydrothermal, range of 160 °C to 240 °C	DP 1–10	[53]
	Pomelo pectin	Electron beam irradiation (125 kGy)	DP 9	[54]
Enzymatic	Olive by-product	Endo-PGase from *Aspergillus aculeatus*, 30 °C, 3 or 24 h	DP 7–15	[55]
	Pectin DM20–34%	Endo-PGases: AtPGLR and AtADPG2 from *Arabidopsis thaliana*, overnight, 25 °C	DP 1–10 or DP 1–9, esterified	[56]
	Pectins DM 24–30% and sugar beet pectins DM 42% DA 31%	PLase from *Verticillium dahliae* (VdPeIB), overnight, 35 °C	DP 2–7, esterified	[57]
	PGA	Endo-PGase *Kluyveromyces fragilis*, 16 h at 37 °C	DP 1–2	[45]
	PGA	Endo-PGase *Aspergillus niger*, 3 h at 30° C	DP 10–15	[58]
	PGA	Endo-PGase *Aspergillus niger*, 3 h	DP 3–18	[59]
	PGA	Endo-PGase AnPG28A from *Aspergillus niger*	DP 2–10	[60]
	Sunflower pectin	Endo-PGase AnPG28A from *Aspergillus niger*, 36 h, 30 °C	DP 2–7, esterified	[24]

**Table 2:** OG production methods, characteristics, mechanisms of action, and protective effects against plant pathogens. Legends: nd: not determined; Endo-PGase: Endo-polygalacturonase; PGA: polygalacturonic acid.

OG Production	DP	DA	DM	Target Pathogens	Host Plant	Plant Responses/ Protective Effect	References
PGA	11	0%	0%	*Botrytis cinerea*	Grapevine	Production of reactive oxygen species, induction of defense genes (chitinases, phenylalanine ammonia-lyase, glucanasesreduction of lesions	[87]
	3–10	0%	0%				[88]
Endo-PGase *A. niger* + PGA	10–15	0%	0%	*Botrytis cinerea*	Tomato	Synthesis of abscisic acid, salicylic acid, jasmonic acid	[58]
Trigalacturonic acid (Sigma T7407, France)	3	0%	0%	*Pectobacterium* *carotovor*	*Arabidopsis thaliana*	Synthesis of salicylic acid, jasmonic acid, reduction of lesions	[16]
OGs (Elicityl, France)	10–15	0%	0%				
Endo-PGase (AnPG28A) + sunflower pectin	2–7	nd	nd	*Rhizoctonia* *solani*	Sugar beet	Overexpression of defense genes encoding peroxidases and superoxide dismutase; reduction of symptoms	[24]
Endo-PGase *A. niger* + PGA	3–18	0%	0%	*Fusarium* *graminearum*	Wheat	Expression of defense genes (PR1, thaumatin, ethylene biosynthesis); reduction of symptoms	[59]
PGA thermal degradation and chemical acetylation	2–25	30%	0%	*Blumeria* *graminis*	Wheat	Reduction of symptoms and induction of enzymes activities (POX, LOX and OXO)	[21]
	2–25	30%	0%	*Aphanomyces* *euteiches*	Pea	Up-regulation of defense genes (PR proteins, catalase); reduction of symptoms	[23]
OGs (Elicityl, France)	10–15	0%	0%	*Botrytis cinerea*	*Arabidopsis thaliana*	Reduction of lesions	[78]
Olive by-product + PGase from *Aspergillus aculeatus*	7–15	nd	nd	*Botrytis cinerea*	*Arabidopsis thaliana*	Induction of Ca^2+^ release and defense genes	[55]
Olive pomace	1–30	nd	nd	*Botrytis cinerea*	*Arabidopsis thaliana*	Expression of defense genes (PAD, WRKY); partial reduction of lesions	[89]
				*Pseudomonas* *syringae*	*Arabidopsis thaliana* and tomato	Expression of defense genes; reduced bacterial growth (20% on *A. thaliana* and 10% on *tomato*)	

## Data Availability

No new data were created or analyzed in this study. Data sharing is not applicable to this article.
